# Beyond Weight Loss: Optimizing GLP-1 Receptor Agonist Use in Children

**DOI:** 10.3390/children12111427

**Published:** 2025-10-22

**Authors:** Hussein Zaitoon, Aimee D. Wauters, Luisa M. Rodriguez, Jane L. Lynch

**Affiliations:** Division of Pediatric Endocrinology and Diabetes, University of Texas Health Science Center, San Antonio, TX 78229, USA; zaitoon@uthscsa.edu (H.Z.); wautersa@uthscsa.edu (A.D.W.); rodriguezl30@uthscsa.edu (L.M.R.)

**Keywords:** GLP-1 receptor agonists, pediatric obesity, liraglutide, semaglutide, body composition

## Abstract

**Background/Objectives**: Glucagon-like peptide-1 receptor agonists (GLP-1RAs) have emerged as a transformative therapy for obesity and type 2 diabetes (T2D) in pediatric populations. This review synthesizes current evidence on efficacy, safety, and knowledge gaps in children and adolescents. **Methods**: A structured review of randomized controlled trials, extension studies, and mechanistic investigations evaluating GLP-1RAs in pediatric obesity and T2D was conducted. Outcomes of interest included body weight, BMI, body composition, glycemic control, and adverse events. **Results**: In adolescents, liraglutide and semaglutide consistently produce clinically meaningful reductions in BMI, body weight, and waist circumference, with modest improvements in systolic blood pressure and minimal effects on lipid levels or HbA1c. A newer trial in children 6 to <12 years showed liraglutide reduced BMI compared with placebo, with GI events consistent with prior safety profiles. Weight loss tends to include both fat and lean components; rapid weight loss may impair muscle strength or bone density if resistance exercise and adequate protein intake are not ensured. Risks include micronutrient gaps, misuse, and uncertain long-term effects on growth and puberty. These important considerations remain largely unaddressed in pediatric studies, and adult data can’t be directly extrapolated to children due to developmental, hormonal, and physiological differences. **Conclusions**: GLP-1 RAs are a promising adjunct to lifestyle therapy for pediatric obesity, but pediatric-specific protocols are needed to safeguard musculoskeletal health, ensure nutritional adequacy, and minimize misuse. Critical gaps remain in pediatric pharmacokinetics, dosing strategies, and long-term developmental safety. Further research is essential to develop evidence-based guidelines for safe and effective pediatric anti-obesity therapy.

## 1. Introduction

Glucagon-like peptide-1 receptor agonists (GLP-1 RAs) are incretin-mimetic agents that promote satiety, lower caloric intake, delay gastric emptying, and enhance pancreatic islet hormone regulation [1,2]. Initial trials of GLP-1 RA focused on treatment for adolescent Type 2 Diabetes (T2D). The landmark longitudinal Treatment Options for Type 2 Diabetes in Adolescents and Youth (TODAY) study design included lifestyle interventions, metformin and a randomized additional rosiglitazone intervention arm [3]. These patients had minimal to no weight loss with relatively rapid beta cell function decline, reduced insulin sensitivity and loss of glycemic control compared to adult onset T2D. The alarming TODAY study outcomes found aggressive progression of diabetes-related complications and emphasized the need to accelerate clinical research of safe and effective therapy alternatives for adolescents with T2D [3,4,5]. GLP-1 RA clinical trials to investigate safety and efficacy in adolescents were prioritized both to study intervention and potential prevention of adolescent T2D in high-risk youth. Exenatide, dulaglutide, liraglutide and semaglutide were the initial GLP-1 RAs approved for clinical trials in adolescents ≥ 10 years of age with T2D. The findings of both improved glycemic control and weight loss along with modest improvements in cardiometabolic risk factors resulted in FDA approvals of GLP-1 RA therapy options [6]. Many gaps exist in understanding the pathophysiology of puberty-related insulin resistance in obese youth to inform effective T2D interventions. Ensuing GLP-1 RA clinical trials in pediatric patients with obesity, when combined with structured lifestyle programs, suggest that early GLP-1 RA therapy may provide even more robust clinically meaningful weight loss and the potential to reduce T2D risk [6,7,8,9].

Currently liraglutide and semaglutide have FDA approval for adolescents ≥12 years of age for specific BMI-based obesity eligibility and are recommended as an adjunct to intensive lifestyle therapy. No GLP-1 RA is yet authorized for obesity in children under 12 years of age. Recent evidence from a 2024 randomized trial in obese participants aged 6 to <12 years demonstrated that liraglutide significantly lowered BMI compared with placebo, with gastrointestinal (GI) effects matching the expected safety profile [9]. Network meta-analyses consistently rank semaglutide as producing the largest reductions in BMI and body weight compared with other GLP-1 RAs in youth populations [7,10]. Individual randomized controlled trials and subsequent pooled analyses confirm that GLP-1 RAs in youth lead to reductions in body weight, BMI (including z-scores), and waist circumference, alongside modest declines in systolic blood pressure. Lipid levels and HbA1c remain largely unchanged in the general pediatric population, though small benefits are observed in those with insulin resistance [10,11,12]. GI adverse events—most commonly nausea and vomiting—are the primary tolerability issue, while mean heart rate increases are minimal and treatment discontinuations are rare [10,11,12]. The 2023 American Academy of Pediatrics (AAP) Clinical Practice Guideline formally recommends considering pharmacotherapy, including GLP-1 RAs, for adolescents ≥12 years with obesity as part of a comprehensive care plan, positioning these agents between lifestyle therapy and bariatric surgery in the treatment algorithm [13]. Safe and effective prescribing recommend gradual dose escalation to minimize GI symptoms, integration with nutrition and behavioral interventions, vigilance for pancreatitis and gallbladder disease, and consideration of access and equity challenges highlighted in recent reviews [14,15].

This narrative review provides a structured synthesis of current evidence on the mechanisms, efficacy, safety, and clinical applications of GLP-1 RAs in children and adolescents. Particular emphasis is placed on research gaps related to body composition, musculoskeletal health, and developmental outcomes. In addition, the review addresses ethical and psychosocial considerations and outlines key priorities to guide future pediatric research and clinical practice.

## 2. Methods

We performed a structured search of PubMed, Embase, and the Cochrane Library (January 2010–March 2025) using terms including GLP-1 RAs, liraglutide, semaglutide, pediatric obesity, adolescents, children, body composition, bone mineral density. Only studies in pediatric populations (≤18 years) were included for primary synthesis.

Inclusion criteria: randomized controlled trials, meta-analyses, and prospective observational studies reporting anthropometric, metabolic, or safety outcomes in children or adolescents. We excluded case reports, letters to the editor and abstracts. Adult studies were included only to fill gaps in pediatric evidence, particularly in musculoskeletal and nutritional outcomes. Study quality was evaluated by sample size, follow-up length, attrition, and outcome definition.

## 3. Results

The structured search identified 1273 records. After duplicate removal and title/abstract screening, 62 articles were reviewed in full. Of these, 11 randomized control studies were chosen for the primary synthesis (Table 1). In addition, relevant meta-analyses and prospective observational studies were examined to complement the interpretation of findings and provide broader clinical context, particularly where pediatric RCT evidence was limited. Targeted searches of adult studies were also conducted in the same databases to address specific gaps in pediatric data.

## 4. Discussion

### 4.1. Mechanisms and Clinical Effects in Youth

GLP-1 RAs reduce body weight predominantly by lowering caloric intake through coordinated central and gastrointestinal mechanisms, with additional contributions to glucose regulation. Within the central nervous system, activation of GLP-1 receptors in the hypothalamus and brainstem enhances satiety pathways, diminishes hedonic eating, and reduces appetite [26]. In the gut, delayed gastric emptying prolongs postprandial fullness and limits meal size. On pancreatic islets, GLP-1 RAs stimulate glucose-dependent insulin release and inhibit glucagon secretion, improving postprandial glucose control without provoking hypoglycemia—an important feature for insulin-resistant youth and those with prediabetes or type 2 diabetes [9,15]. These physiological effects are reflected in clinical outcomes: pediatric randomized controlled trials report consistent reductions in BMI/BMI z-score (Table 1), absolute weight, and waist circumference, often accompanied by modest improvements in systolic blood pressure, with little to no change in lipid levels or HbA1c in otherwise normoglycemic participants [10,11,12]. Meta-analyses confirm these findings and note predictable, mechanism-related adverse events, primarily gastrointestinal discomfort and small increases in heart rate [7,10,11,12]. In children aged 6 to <12 years, liraglutide has demonstrated BMI reductions compared with placebo, indicating that appetite suppression and slowed gastric emptying are operative even in younger age groups [9].

Beyond their effects on appetite and gastric emptying, GLP-1 RAs exert direct endocrine actions on pancreatic islets that contribute to metabolic regulation in pediatric populations. By binding to GLP-1 receptors on β-cells, these agents potentiate glucose-dependent insulin secretion, improving first- and second-phase insulin responses during meals without driving hypoglycemia—an especially important consideration for children with preserved β-cell function or insulin resistance rather than absolute insulin deficiency [19,27,28]. Concurrently, α-cell GLP-1 receptor activation suppresses inappropriate glucagon secretion in the postprandial state, reducing hepatic glucose output and attenuating glycemic excursions [29]. In youth with impaired glucose tolerance or early type 2 diabetes, these dual effects can improve oral glucose tolerance profiles and reduce glycemic variability, thereby mitigating the β-cell stress imposed by chronic hyperglycemia [30,31]. Fluctuations in gastric emptying and central appetite regulation during adolescence, influenced by the hormonal shifts of puberty, may affect both the potency and timing of GLP-1 receptor agonist treatment responses. Although pediatric cohort studies are lacking, animal research indicates potential impacts on the hypothalamic-pituitary-gonadal axis [32]. These developmental and physiological distinctions highlight the necessity for tailored dosing strategies and careful response monitoring in youth to assess for unique metabolic differences from adult modeling.

### 4.2. Current Approvals and Clinical Efficacy

Liraglutide was approved in 2019 for pediatric T2D in patients ≥10 years; semaglutide received FDA approval in 2022 for adolescent obesity (12–17 years). Clinical trials demonstrated that liraglutide achieved −4.5% [95% CI, −7.17 to −1.84%] mean weight loss and semaglutide achieved −17.7% [95% CI, −21.8 to −13.7%] mean weight loss, outperforming lifestyle intervention alone [21,24]. Beyond weight control, adult trials have shown improved insulin resistance-related comorbidities such as metabolic dysfunction-associated steatotic liver disease, dyslipidemia, and polycystic ovary syndrome [33,34,35]. Semaglutide has also been approved for reducing major cardiovascular events in adults with overweight or obesity [36]. Both drugs require subcutaneous injection—liraglutide daily and semaglutide weekly—with gradual dose escalation to minimize side effects. Nausea and vomiting are the most common adverse events, leading to discontinuation in 5–10% of adolescent trial participants [21,24]. Tolerability may improve with slow titration, dietary adjustments, or short-term antiemetic use. Serious risks are rare but can include pancreatitis, cholecystitis, and, in preclinical studies, potential C-cell tumors—contraindicating use in individuals with relevant personal or family history [21,24,37]. These medications are not recommended during pregnancy, as data remain limited and animal studies raise concerns about fetal growth [38,39].

Emerging agent adult data show potential to enhance outcomes further. Tirzepatide, a dual GLP-1/GIP agonist, has produced up to 19.7% weight loss and reduced T2D progression in adults; after 17 weeks risk of T2D (2.4% tirzepatide group and 13.7% placebo group, hazard ratio, 0.12; 95% CI, 0.1 to 0.2; *p* < 0.001) [40]. Triple agonists like retatrutide—targeting GLP-1, GIP, and glucagon receptors—have achieved up to 24.2% weight loss in adults (12-mg group), signaling a new generation of anti-obesity pharmacotherapy [41].

### 4.3. Nutritional and Lifestyle Considerations

Adult studies with GLP-1 RAs for obesity often cause substantial appetite suppression, reducing caloric intake by 16–39% [42]. When daily intake falls below approximately 1200 kcal for adult females or 1800 kcal for adult males, the risk of inadequate micronutrient intake increases sharply [43]. Commonly affected nutrients include iron, calcium, magnesium, zinc, and vitamins A, D, E, K, B1, B12, and C [44]. Deficiencies may manifest as fatigue beyond that expected from weight loss, marked hair shedding, dry or itchy skin, muscle weakness, impaired wound healing, or easy bruising [45]. Gastrointestinal side effects of GLP-1 RAs can further impair absorption, exacerbating pre-existing deficiencies or triggering new ones. Many individuals with obesity already present with suboptimal diet quality—often high in ultra-processed foods or shaped by restrictive eating patterns—predisposing them to deficiencies before pharmacologic intervention. Additionally, obesity-related alterations in nutrient absorption, metabolism, and utilization can further compromise status [46]. These factors underscore the need for intentional dietary planning to maximize nutrient density within reduced caloric limits [44].

### 4.4. Body Composition, Muscle, and Bone Health

GLP-1Ras-induced weight loss typically involves reductions in both adipose and lean tissue [47,48]. In the STEP 1 trial, adults receiving semaglutide lost a mean of 15.3 kg at 68 weeks compared with 2.6 kg with placebo (treatment difference, −12.7 kg; 95% CI, −13.7 to −11.7), dual-energy X-ray absorptiometry (DXA) substudy data showed fat mass reductions of 8.3 kg (62%) versus lean mass reductions of 5.3 kg (38%), of which skeletal muscle loss accounted for ~20% of total weight lost [49]. In SURMOUNT 1, Tirzepatide reduced total lean mass by −10.9% versus −2.6% with placebo, an estimated treatment difference of −8.3% (95% CI, −10.6 to −6.1) [50]. Without resistance training, muscle loss generally accounts for approximately 10–15% of weight reduction in women and 20–25% in men [51]. The magnitude of loss is influenced by calorie restriction severity, weight-loss rate, protein adequacy, and engagement in strength training [52]. Preclinical and physiological data indicate increased skeletal muscle microvascular blood flow and activation of anti-atrophic pathways, which may help preserve function despite modest volume loss [47,53,54]. Clinically, combining GLP-1RAs with resistance exercise and adequate protein intake is essential to protect muscle strength and metabolic health. In youth, the musculoskeletal effects of GLP-1 RA are hypothesized to largely be parallel to the adult patterns but must be interpreted with caution. Musculoskeletal changes must be explored in the context of adolescent secretion of puberty and growth-related hormones and linear growth that exaggerate the BMI score reductions during concurrent weight loss and improved insulin sensitivity.

Adults with rapid weight loss (≥14% within 3–4 months) are at risk for measurable declines in bone mineral density (BMD) [55], whereas slower, moderate loss is more likely to preserve bone integrity [56]. Bone loss risk is shaped by baseline weight, age, sex, physical activity, protein intake, and rate of weight change, with older individuals and women at highest vulnerability [57]. Weight cycling—intermittent GLP-1RA use with subsequent weight regain—may compound both muscle and bone losses, potentially leading to sarcopenic obesity. Mechanistically, GLP-1 signaling may stimulate osteoblast activity and inhibit bone resorption, but human data are mixed and confounded by the effects of weight loss, some studies show BMD reductions at weight-bearing sites proportional to weight loss but no deterioration in bone quality or rise in fragility fractures, highlighting mechanical unloading as the primary driver rather than direct drug toxicity [8,12,57,58,59]. In adolescents receiving GLP-1 RAs for obesity, randomized trials remain underpowered for skeletal endpoints. Until more evidence emerges, ensuring adequate calcium and vitamin D intake, promoting regular weight-bearing exercise, and monitoring bone health is prudent—particularly in cases of substantial weight loss.

Obesity prevalence is often influenced by poor dietary habits, inadequate physical activity, and structural lifestyle barriers, with dietary composition playing a key role. While genetic factors modify individual risk, population trends are largely driven by cultural and environmental changes, as well as iatrogenic contributors [60]. Evidence-based adult guidelines recommend intensive, multicomponent behavioral interventions—including nutrition, physical activity, self-monitoring, barrier identification, and relapse prevention—for weight reduction and maintenance [61,62]. Medical nutrition therapy, delivered by a registered dietitian nutritionist, should be a key component of any weight loss plan for children. Individualized nutrition recommendations should consider patients’ current behaviors, preferences, food access, and cultural & social backgrounds. For optimal weight loss, interventional visits should be frequent (≥16 sessions in 6 months) [63]. Frequent visits may be particularly important in children utilizing GLP-1 therapy, as rapid weight loss could potentially lead to nutrient deficiency. Despite strong guideline support, comprehensive lifestyle counseling before or alongside pharmacologic therapies like GLP-1 RAs remains underutilized in practice. Barriers often include short clinical visits, limited access to structured programs, inadequate reimbursement for nutrition counseling, and insufficient practitioner training in lifestyle medicine. Many patients start GLP-1 RAs therapy without prior nutrition or behavioral guidance, limiting long-term efficacy and weight maintenance [64,65]. The recommended approach should ensure patient-centered care that addresses social determinants of health, creates culturally tailored diet and activity plans, and involves multidisciplinary support. Baseline nutritional and medical assessments should evaluate dietary habits, eating disorders, sarcopenia risk, comorbidities, and emotional triggers for overeating. Gradual dose titration, small frequent meals, hydration, and targeted dietary modifications can improve tolerability. For constipation, gradual fiber increase, magnesium supplementation, and osmotic laxatives may help, whereas diarrhea can be addressed with dietary adjustments, fiber supplementation, or anti-diarrheal agents. Clinicians should emphasize that GLP-1 RAs are adjuncts—not replacements—for long-term nutrition and lifestyle change, with counseling focused on sustaining health improvements beyond weight loss. This integrated approach optimizes GLP-1 RAs benefits, minimizes risks, and supports durable obesity management.

### 4.5. Monitoring and Clinical Implementation

Evaluating body composition—including lean mass, fat mass, muscle function, and BMD—is a critical component of care that merits further research and outcome data analysis for patients receiving GLP-1 RAs for the spectrum of impaired glucose tolerance, early T2D, T2D and obesity. These agents promote significant weight loss that, while predominantly fat mass, consistently includes a meaningful proportion of lean mass—often 15–25%—as shown in clinical trials and meta-analyses [54,66,67,68]. Rapid or substantial weight loss can also lower BMD, with effects more pronounced in the absence of exercise. The Obesity Society advises routine monitoring and integration of physical activity interventions to counteract these risks [65].

Evidence-based data are lacking in youth who are still developing peak muscle and bone mass regarding optimal protein intake and safe structured resistance exercise. Determinates of relative lean muscle and fat loss to inform the impact of GLP-1 RA therapy on physical function, metabolic health, and long-term cardiometabolic outcomes in youth are needed [47,68,69]. Early identification of disproportionate lean or bone loss—via standardized assessments—could potentially guide timely adjustments to optimize nutrition and exercise strategies, safeguarding growth and long-term health in pediatric patients. For pediatric populations on GLP-1 RA therapy, the most reliable modalities for tracking body composition are DXA, bioelectrical impedance analysis (BIA), and, in select cases, air displacement plethysmography. DXA remains the gold standard for quantifying lean and fat mass as well as BMD, with annual or biennial use recommended when feasible, though accessibility and programming requirements may be limiting factors. BIA offers a more practical and cost-effective option for frequent point-of-care monitoring, albeit with lower precision compared to DXA. Air displacement plethysmography serves as a valuable alternative for patients with implanted devices who cannot undergo BIA, though it requires specialized facilities and trained personnel. Currently, no imaging techniques are validated for routine assessment of pediatric muscle quality; therefore, functional measures such as handgrip strength, sit-to-stand testing, or tailored strength evaluations by an exercise physiologist are recommended, despite their lower sensitivity in children. The 2025 joint advisory from The Obesity Society highlights that these assessments should be paired with structured resistance training and sufficient dietary protein to maintain muscle and bone integrity during GLP-1-induced weight loss [68]. This integrated approach ensures that pharmacologic weight reduction is achieved without compromising the musculoskeletal development essential for healthy growth and long-term well-being.

### 4.6. Ethical and Psychosocial Considerations

The rapid expansion of GLP-1 RAs into pediatric care raises pressing concerns about unintended consequences. The successes in managing body weight and improving diabetes may inadvertently accelerate GLP-1 RAs widespread use, both with and without medical supervision. The development of oral formulations [70] will likely increase accessibility, compounding risks during critical periods of growth when appetite suppression, altered energy balance, and fatigue could disrupt hormonal and developmental pathways. Potential harms range from impacts on bone mineralization and long-term growth [71] to heightened vulnerability in groups with eating disorders, body-image concerns, or participation in weight-sensitive sports. Moreover, pediatricians often lack specialized training in obesity and fitness management, which may lead to inappropriate prescribing patterns in populations already at elevated metabolic risk.

The adolescent years present a perfect storm for potential misuse. Risk-taking tendencies [72], high exposure to body-image pressures via social media [73], and the lingering effects of pandemic-related lifestyle disruptions have amplified eating disorder prevalence [74]. Coupled with awareness—fueled by both popular media and illicit online drug markets [75]—GLP-1 RAs could attract misuse among youth in competitive sports, aesthetic disciplines, or those with distorted self-perception of weight [76,77]. The emergence of counterfeit drugs [78] and the commercialization of weight control add further hazards. These patterns may divert focus from proven lifestyle-based approaches, which have consistently shown superior long-term effectiveness compared to medication in adults [79], yet are still underutilized in treating children.

Beyond misuse, key pharmacological and socioeconomic gaps remain. Pediatric physiology differs significantly from adults [80], limiting the validity of direct data extrapolation—even where studies on drugs like liraglutide exist [81]. The appropriate dosing, safety considerations, and metabolic effects of these medications throughout childhood and adolescence remain poorly characterized, and pediatric-specific formulations tend to be more expensive [82] and may increase the risk of prolonged reliance on treatment. Effective use is likely to require concurrent lifestyle modification [83], yet most current interventions fail to measure diet and activity rigorously or move beyond the flawed reliance on BMI as a marker of adiposity [84]. The result is a fragmented approach that risks prioritizing drug therapy over sustainable health behavior change, particularly in communities already facing health disparities. A crucial and practical distinction remains between FDA approval of drug use based on safety and efficacy and the reality of limitations in insurance coverage policies which often thwart actual authorization and reimbursement for prescribed medications. Furthermore, insurance coverage is often unstable in youth and is at risk to drop at age 18 years. To counter these risks, recommended actions include building multidisciplinary teams and community leaders to address knowledge gaps; advancing translational bioethics for pediatric pharmacotherapy [85]; leveraging school-based physical fitness testing [86] to identify at-risk populations; and working with regulatory bodies to modernize pediatric lifestyle guidelines. Efforts should also target the training of both clinical trialists and primary care pediatricians [87], alongside public education for teachers, parents, and children about safe and appropriate GLP-1 RA use. Such coordinated measures may help harness the benefits of these potent medications while minimizing long-term harm during the most formative years of growth and development.

From a practical standpoint, while the need for musculoskeletal monitoring and multidisciplinary care is clear, it is important to emphasize that no evidence-based clinical recommendations can yet be made for pediatric use. Current pediatric trials are short-term, underpowered, and primarily designed for weight and safety outcomes rather than musculoskeletal endpoints. Accordingly, parameters such as the optimal frequency of DXA or BIA assessments, target protein intake, or the type and intensity of exercise have not been established in children or adolescents receiving GLP-1 RAs.

Nevertheless, interim clinical practice can be informed by general pediatric obesity and endocrinology guidelines, which emphasize maintaining adequate dietary protein and calcium intake [88,89], ensuring regular weight-bearing physical activity [90], and providing individualized nutrition and behavioral support. Until robust, long-term pediatric data are available, clinicians should apply these principles cautiously, within a multidisciplinary framework that includes endocrinology, nutrition, and physical therapy, and interpret any body composition changes in light of growth, puberty, and developmental status.

## 5. Conclusions

GLP-1 RAs offer a new therapeutic option for pediatric obesity and early type 2 diabetes, producing meaningful weight reduction and metabolic improvement. However, the current evidence base in children remains limited—characterized by short duration, small sample sizes, and heterogeneous outcomes. Major gaps include the absence of long-term safety and growth data, lack of pediatric-specific dosing and pharmacokinetic studies, and lack of recommendation for evaluation of body composition, micronutrient status, and psychosocial effects. Addressing these gaps through rigorous, multidisciplinary, and age-appropriate research is essential to guide the safe and effective use of GLP-1 therapies in children and adolescents.

## Figures and Tables

**Table 1:** Summary of Randomized Control Studies Evaluating GLP-1 Receptor Agonists in Children and Adolescents.

Study (Year)	Agent/Comparator	Population (Age, Condition)	*n* = Study Group w/o Controls	Duration	Main Efficacy Outcomes
Kelly et al., 2013 [16]	Exenatide twice daily vs. placebo	12–<18 y, obesity	*n* = 13	12 weeks	−3.26 kg weight reduction; −1.13 kg/m^2^ BMI; −2.70% mean BMI (%) change
Klein et al., 2014 [17]	Liraglutide 1.8 mg daily vs. placebo	12–<18 y, type 2 diabetes	*n* = 14	5 weeks	HbA1c −0.86%; body weight remained stable
Danne et al., 2017 [18]	Liraglutide 3.0 mg daily vs. placebo	12–<18 y, obesity	*n* = 14	5 weeks	Results NS: −2.55 kg weight reduction; −0.12 kg/m^2^ BMI z-score
Tamborlane et al., 2019 [19]	Liraglutide 1.8 mg daily vs. placebo	10–<17 y, type 2 diabetes	*n* = 66	52 weeks	HbA1c −0.50%; −0.34 kg/m^2^ BMI z-score; improved postprandial glucose
Mastrandrea et al., 2019 [20]	Liraglutide daily 3.0 mg vs. placebo	7–<11 y, obesity	*n* = 16	Up to 13 weeks	−0.3 kg/m^2^ BMI z-score;−0.52 kg weight reduction (NS)
Kelly et al., 2020 [21]	Liraglutide 3.0 mg daily vs. placebo	12–<18 y, obesity	*n* = 125	56 weeks	−5.01% body weight; −0.22 kg/m^2^ BMI z-score; 43.3% ≥5% BMI reduction
Weghuber et al., 2020 [22]	Exenatide 2.0 mg weekly vs. placebo	12–<18 y, obesity	*n* = 22	24 weeks	−3.0 kg body weight; −0.09 kg/m^2^ BMI z-score
Fox et al., 2022 [23]	Exenatide XR weekly 2.0 mg vs. placebo	10–<18 y, obesity	*n* = 33	52 weeks	−2.7 mean BMI (kg/m^2^) reduction; −4.6% mean BMI (%) change
Weghuber et al., 2022 [24]	Semaglutide 2.4 mg weekly vs. placebo	12–<18 y, obesity	*n* = 201	68 weeks	−16.1% body weight, −1.1 kg/m^2^ BMI z-score; 73% achieved ≥5% weight reduction
Arslanian et al., 2022 [25]	Dulaglutide 0.75 mg or 1.5 mg weekly vs. placebo	10–<18 y, type 2 diabetes	*n* = 103 (both doses)	26 weeks	HbA1c reduction (−0.6% to −0.9% dose related); stable weight; improved fasting glucose
Fox et al., 2025 [9]	Liraglutide 3.0 mg daily vs. placebo	6–<12 y, obesity	*n* = 56	56 weeks	−5.8% change in BMI; −0.7 kg/m^2^ BMI z-score; 46% ≥5% BMI reduction

BMI = body mass index; HbA1c = glycated hemoglobin; NS = not significant. All studies included pediatric or adolescent participants with obesity or type 2 diabetes. Sample sizes (*n*) represent the number of participants in the active treatment arm, excluding placebo groups. Efficacy outcomes are reported as changes from baseline unless otherwise specified.

## Data Availability

No new data were created or analyzed in this study. Data sharing is not applicable to this article.

## Abbreviations

GLP-1 RAs	Glucagon-like peptide-1 receptor agonists
BMI	Body mass index
BMD	Bone mineral density
GI	Gastrointestinal
T2D	Type 2 diabetes
DXA	Dual-energy X-ray absorptiometry
BIA	Bioelectrical impedance analysis
